# Role of Type II Protein Arginine Methyltransferase 5 in the Regulation of Circadian *Per1* Gene

**DOI:** 10.1371/journal.pone.0048152

**Published:** 2012-10-25

**Authors:** Jungtae Na, Kwanghyun Lee, Hwan-Gon Kim, Jee-Yoon Shin, Wonho Na, Hayan Jeong, Jong-Woo Lee, Sehyung Cho, Won-Sun Kim, Bong-Gun Ju

**Affiliations:** 1 Department of Life Science, Sogang University, Seoul, Korea; 2 Department of Physiology, Kyung Hee University School of Medicine, Seoul, Korea; Karlsruhe Institute of Technology, Germany

## Abstract

Circadian clocks are the endogenous oscillators that regulate rhythmic physiological and behavioral changes to correspond to daily light-dark cycles. Molecular dissections have revealed that transcriptional feedback loops of the circadian clock genes drive the molecular oscillation, in which PER/CRY complexes inhibit the transcriptional activity of the CLOCK/BMAL1 heterodimer to constitute a negative feedback loop. In this study, we identified the type II protein arginine methyltransferase 5 (PRMT5) as an interacting molecule of CRY1. Although the *Prmt5* gene was constitutively expressed, increased interaction of PRMT5 with CRY1 was observed when the *Per1* gene was repressed both in synchronized mouse liver and NIH3T3 cells. Moreover, rhythmic recruitment of PRMT5 and CRY1 to the *Per1* gene promoter was found to be associated with an increased level of histone H4R3 dimethylation and *Per1* gene repression. Consistently, decreased histone H4R3 dimethylation and altered rhythmic *Per1* gene expression were observed in *Prmt5*-depleted cells. Taken together, these findings provide an insight into the link between histone arginine methylation by PRMT5 and transcriptional regulation of the circadian *Per1* gene.

## Introduction

Circadian clocks are the endogenous oscillators that drive metabolic, physiological, and behavioral rhythms with an intrinsic period of approximately 24 hours [1]. The circadian clocks are entrained to day-night cycles generated by the rotation of the Earth. In mammals, the suprachiasmatic nucleus (SCN) in the anterior hypothalamus functions as a central clock, which orchestrates peripheral clocks present in almost every tissue, even in cultured cells [2], [3], [4], [5]. At the molecular level, transcriptional/translational feedback loops underlie the mammalian circadian clocks that give rise to molecular oscillation through the action of transcriptional factors such as CLOCK/BMAL1 transcriptional activators and PER/CRY transcriptional repressors [6], [7]. The CLOCK/BMAL1 heterodimer transactivates clock genes including *Pers* (*Per1, Per2, Per3*) and *Crys* (*Cry1, Cry2*) via binding to the E-box on their promoter region. The resulting PER/CRY complexes translocate into the nucleus with a timed delay, and then inhibit the CLOCK/BMAL1-mediated transcriptional activation.

CRYs are flavin/pterin-containing proteins and were initially identified as plant blue-light receptors due to their structural similarity to photolyase, which repairs DNA photoproducts in response to UV irradiation [8]. Although mammalian CRYs have been reported to have no direct DNA repair activity, it has been suggested that mammalian CRYs participate in DNA repair through transcriptional regulation of the *Xpa* gene, a component of nucleotide excision repair, in a circadian rhythm-dependent manner [9]. Genetically modified mice with two *Crys* inactivated are completely arrhythmic, indicating that CRYs are critical components of the central circadian pacemaker [10], [11].

Accumulating evidence suggests that post-transcriptional modifications of CRY play an important role in circadian rhythm regulation. For example, casein kinase I phosphorylates PERs, which associates with CRYs, leading to translocation of the PERs/CRYs complexes and inhibition of CLOCK/BMAL1-driven transcription [12], [13], [14], [15]. CRY1 undergoes ubiquitination by F-box and leucine-rich repeat protein 3 (FBXL3), which results in its subsequent degradation [16], [17], [18]. Recent study suggests that adenosine monophosphate-activated protein kinase (AMPK) phosphorylates CRY1 and destabilizes it in response to nutrient signals in the mouse liver [19]. It has also been reported that CRY1 inhibits the CLOCK/BMAL1-mediated transcriptional activation through regulation of histone modifications. In fact, it has been shown that CRY1 negatively regulates *Per1* gene expression by recruiting histone deacetylases (HDACs) and mSin3B [20]. Dimethylation of histone H3K9 and recruitment of HP1α to the *Dbp* (*albumin D site-binding protein*) gene promoter might be the general mechanism for CRY-mediated transcription repression [21].

In this study, we demonstrate that type II protein arginine methyltransferase 5 (PRMT5), which is known as a transcriptional corepressor due to its dimethylation activity on histone H4R3, interacts with CRY1 in the synchronized mouse liver and NIH3T3 cells. In contrast to rhythmic expression of *Cry1* and *Per1* genes, non-rhythmic expression of the *Prmt5* gene was observed. However, rhythmic recruitment of CRY1 and PRMT5 to the *Per1* gene promoter coincides with the rhythmic dimethylation of histone H4R3 and *Per1* gene repression. Consistently, we found decreased H4R3 dimethylation and alteration of rhythmic *Per1* gene expression in *Prmt5*-depleted cells.

## Results

### CRY1 interacts with PRMT5

**Figure 1:**
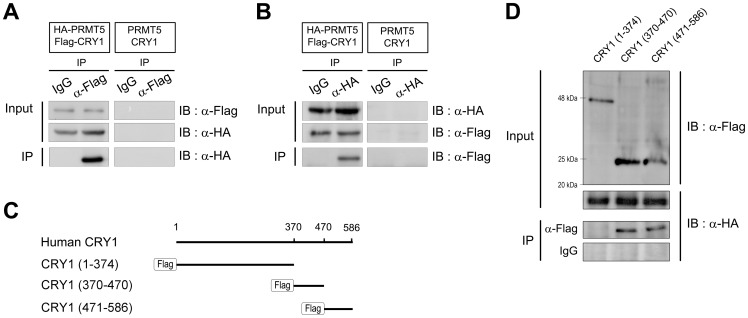
CRY1 interacts with PRMT5. (A, B) The exogenously expressed HA-tagged PRMT5 interacts with Flag-tagged CRY1 in 293T cells (left panel). To avoid nonspecific immunoprecipitation by anti-Flag or anti-HA antibodies, immunoprecipitation/Western blot analysis was performed using overexpressed untagged PRMT5 and CRY1 (right panel). (A) The lysates were immunoprecipitated with the anti-Flag antibody and immunoblotted with the anti-HA antibody or (B) immunoprecipitated with the anti-HA antibody and immunoblotted with the anti-Flag antibody. (C) Schematic diagram showing the full-length CRY1 and its deletion mutants. (D) PRMT5 interacts with CRY1 (aa 370 to 470) and CRY1 (aa 471 to 586), respectively. The lysates from the 293T cell overexpressing deletion fragments of CRY1 and HA-tagged PRMT5 were immunoprecipitated with the anti-Flag antibody and immunoblotted with the anti-HA antibody. IgG was used as the negative control.

To identify possible histone-modifying enzymes involved in CRY1-mediated circadian clock gene regulation, we first immunoprecipitated CRY1-associated proteins from 293T cells overexpressing Flag-tagged CRY1 and performed liquid chromatography-tandem mass spectrometry (Table S1). Interestingly, type II protein arginine methyltransferase 5 (PRMT5) was identified as a CRY1-interacting partner. To verify the interaction between CRY1 and PRMT5, we overexpressed Flag-tagged CRY1 and HA-tagged PRMT5 in 293T cells. Cell extracts were immunoprecipitated with the anti-Flag antibody, followed by Western blot analysis with the anti-HA antibody. While immunoprecipitation with control IgG showed no specific PRMT5 interaction, the overexpressed CRY1 clearly interacted with PRMT5 *in vivo* (Figure 1A). When cell extracts were reciprocally immunoprecipitated with the anti-HA antibody and Western blot analysis with the anti-Flag antibody was performed, a significant interaction of PRMT5 with CRY1 was observed (Figure 1B). To eliminate the possibility that overexpressed proteins are nonspecifically immunoprecipitated by anti-Flag or anti-HA antibodies, we performed immunoprecipitation/Western blot analysis using overexpressed untagged PRMT5 and CRY1 in 293T cells (Figures 1A, 1B, S1A, and S1B). We also tested the interaction between CRY1 and NF-κB (p65) as a negative control (Figure S1C).

We further investigated the interaction region of CRY1 with PRMT5 using a variety of deletion mutants, i.e., Flag-tagged CRY1 (aa 1 to 374), Flag-tagged CRY1 (aa 370 to 470), and Flag-tagged CRY1 (aa 471 to 586) (Figure 1C). HA-tagged PRMT5 equally interacted with Flag-tagged CRY1 (aa 370 to 470) and Flag-tagged CRY1 (aa 471 to 586), respectively (Figure 1D). However, our results suggest that CRY1 (aa 471 to 586) was less strongly associated with PRMT5, since expression of Flag-tagged CRY1 (aa 471 to 586) was weak or unstable compared to that of Flag-tagged CRY1 (aa 370 to 470) in 293T cells (Figures 1D, S1D, and S1E). Interestingly, we detected each homodimeric form of Flag-tagged CRY1 (aa 370 to 470) and Flag-tagged CRY1 (aa 471 to 586) in the Western blot (Figures 1D, S1D, and S1E). This observation was supported by the dissociation of monomer forms when the cell extract preparation and SDS-PAGE were performed in the presence of 4 M urea (Figure S1E).

### PRMT5 acts as a transcriptional repressor of the *Per1* gene

**Figure 2:**
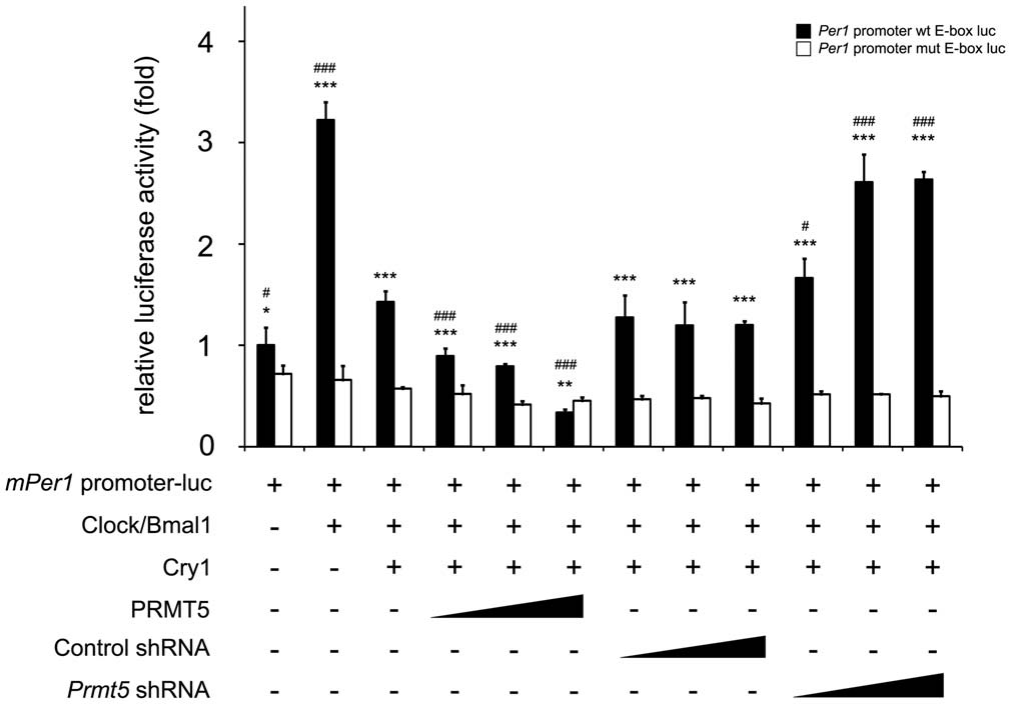
PRMT5 acts as a transcriptional repressor of the *Per1* promoter-reporter gene containing the E-box. Overexpression of PRMT5 enhances CRY1-mediated repression of the E-box containing reporter gene. NIH3T3 cells were transiently transfected with the *Per1* gene promoter-driven firefly luciferase reporter vector (20 ng) in conjunction with a control *thymidine kinase* promoter-driven Renilla luciferase vector. Expression vectors for Myc-tagged CLOCK (200 ng), Myc-tagged BMAL1 (200 ng), Flag-tagged CRY1 (10 ng), HA-tagged PRMT5 (100, 200, 400 ng), PRMT5 shRNA (100, 200, 400 ng), and control shRNA (100, 200, 400 ng) were transfected in combination. As a negative control, the *Per1* promoter containing the mutated E-box-driven firefly luciferase reporter vector was used. Data are presented as mean ± S.E.M. of the three biological replicates with two cell plates analyzed for each group. A paired t-test was used for two group comparison. When luciferase activity of the wt E-box reporter was compared to that of the mut E-box reporter, significance values were * *P*≤0.05, ** *P*≤0.01, *** *P*≤0.005. ^#^ *P*≤0.05, ^##^ *P*≤0.01, and ^###^ *P*≤0.005 compared to the third column in the group of the wt E-box reporters.

PRMT5 is known to be a transcriptional corepressor due to its activity on the dimethylation of histone H3R8 and H4R3 [22]. In addition, we isolated PRMT5 as an associated protein of the CRY1 transcriptional repressor. Therefore, we tested whether it has a transcriptional inhibitory effect on CLOCK/BMAL1-driven circadian clock gene regulation using the *Per1* gene promoter luciferase-reporter containing E-box (Figure 2). While *Per1* promoter activity increased in CLOCK/BMAL1 co-expressed 293T cells, overexpression of PRMT5 augmented the transcriptional repression activity of CRY1. Depletion of *Prmt5* by shRNA transfection abolished CRY1-mediated inhibition of *Per1* promoter activity. Significant inhibition of PRMT5-mediated promoter activity was not observed when the *Per1* promoter luciferase-reporter containing mutated E-box was used as a negative control (Figure 2). These results suggest that PRMT5 may act as a transcriptional corepressor of CRY1 in CLOCK/BMAL1-mediated circadian clock gene regulation.

### PRMT5 is nonrhythmically expressed but rhythmically interacts with CRY1

**Figure 3:**
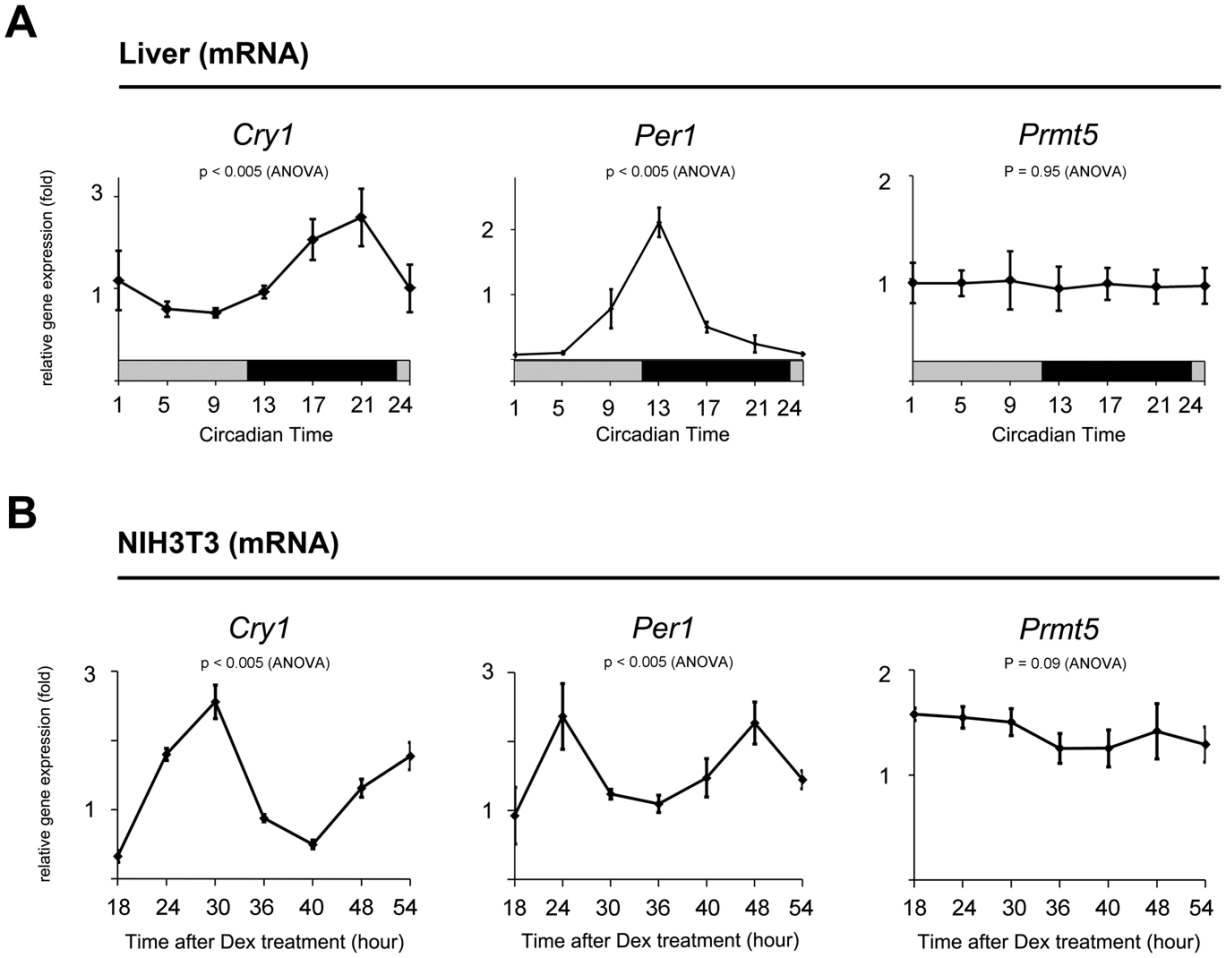
*Prmt5* is non-rhythmically expressed. At the indicated times, total RNA was isolated from (A) mouse livers harvested during the circadian cycle or (B) NIH3T3 cells that were synchronized with 100 nM dexamethasone for 2 hours. Transcripts of *Cry1, Per1*, and *Prmt5* were determined by quantitative PCR. The *Gapdh* transcript was used for normalization. Data are presented as mean ± S.E.M. of the three biological replicates with two mice or two cell plates analyzed for each group. Analysis of variance (ANOVA) was used for multiple comparisons.

In order to investigate whether the *Prmt5* gene is rhythmically expressed, we performed quantitative PCR using mouse liver samples harvested during the circadian cycle. The expression level of the *Cry1* gene peaked at CT (circadian time) 21, whereas that of the *Per1* gene peaked at CT 13, indicating that these genes are rhythmically expressed in the mouse liver (Figure 3A). However, we did not observe clear rhythmic *Prmt5* gene expression (Figure 3A). To further confirm this result, we also examined the expression pattern of the *Prmt5* gene using a synchronized NIH3T3 cell model treated with dexamethasone. Similarly, nonrhythmic expression of the *Prmt5* gene was evident, while the *Cry1* and *Per1* genes were expressed rhythmically in the synchronized NIH3T3 cells (Figure 3B).

**Figure 4:**
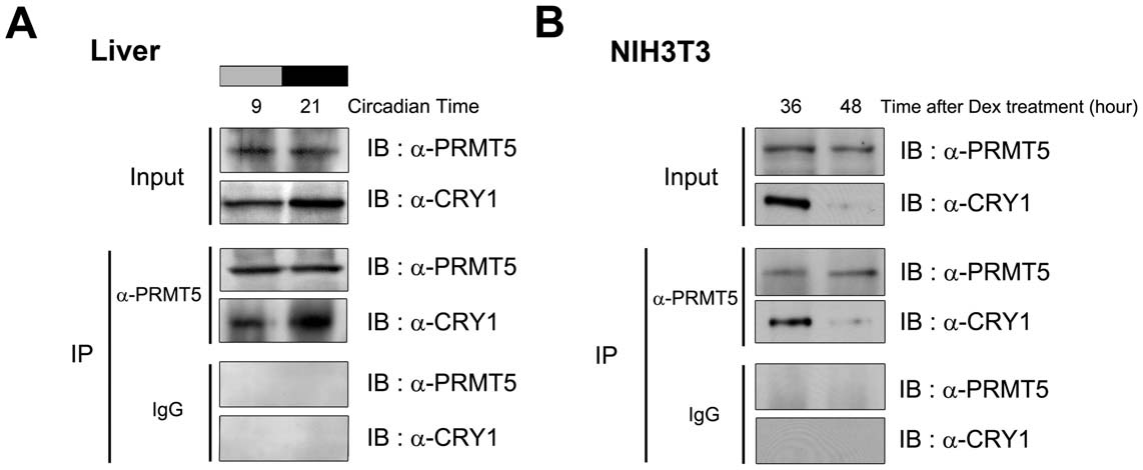
Rhythmic interaction of PRMT5 with CRY1. At the indicated times, nuclear extracts from (A) mouse livers harvested during the circadian cycle or (B) synchronized NIH3T3 cells were immunoprecipitated with the anti-PRMT5 antibody and immunoblotted with the anti-CRY1 or anti-PRMT5 antibody. IgG was used as the negative control.

Given that the *Prmt5* gene was not rhythmically expressed, we investigated whether PRMT5 interacts rhythmically with CRY1 when *Per1* gene expression was either activated or repressed in the mouse liver. We performed immunoprecipitation/Western blot analysis using nuclear extracts with the anti-PRMT5 and CRY1 antibodies, which were confirmed their specificity (Figures S2A and S2B). The endogenous interaction of PRMT5 with CRY1 increased when *Per1* gene expression was repressed (CT 21) (Figures 3A and 4A). In contrast, when *Per1* gene expression was activated (CT 9), a weak interaction of PRMT5 with CRY1 was observed. To confirm rhythmic interaction of PRMT5 and CRY1 in the synchronized NIH3T3 cells, we performed the same immunoprecipitation/Western blot analysis. As expected, the interaction of PRMT5 with CRY1 increased when the expression level of the *Per1* gene was low at 36 hours after synchronization (Figures 3B and 4B). When the gene expression level of *Per1* reached a peak at 48 hours after synchronization, the interaction of PRMT5 with CRY1 significantly decreased. The specificity in the interaction was further confirmed by immunoprecipitation/Western blot analysis using *Cry1*-depleted or *Prmt5*-depleted NIH3T3 cells (Figures S1F and S1G). In light of the fact that FBXL3-mediated degradation of CRY1 occurred when the *Per1* gene was activated [16], our results suggest that rhythmic interaction between PRMT5 and CRY1 could be the product of rhythmic degradation of CRY1 in the synchronized mouse liver and NIH3T3 cells.

### Rhythmic recruitment of PRMT5 to the *Per1* gene promoter

**Figure 5:**
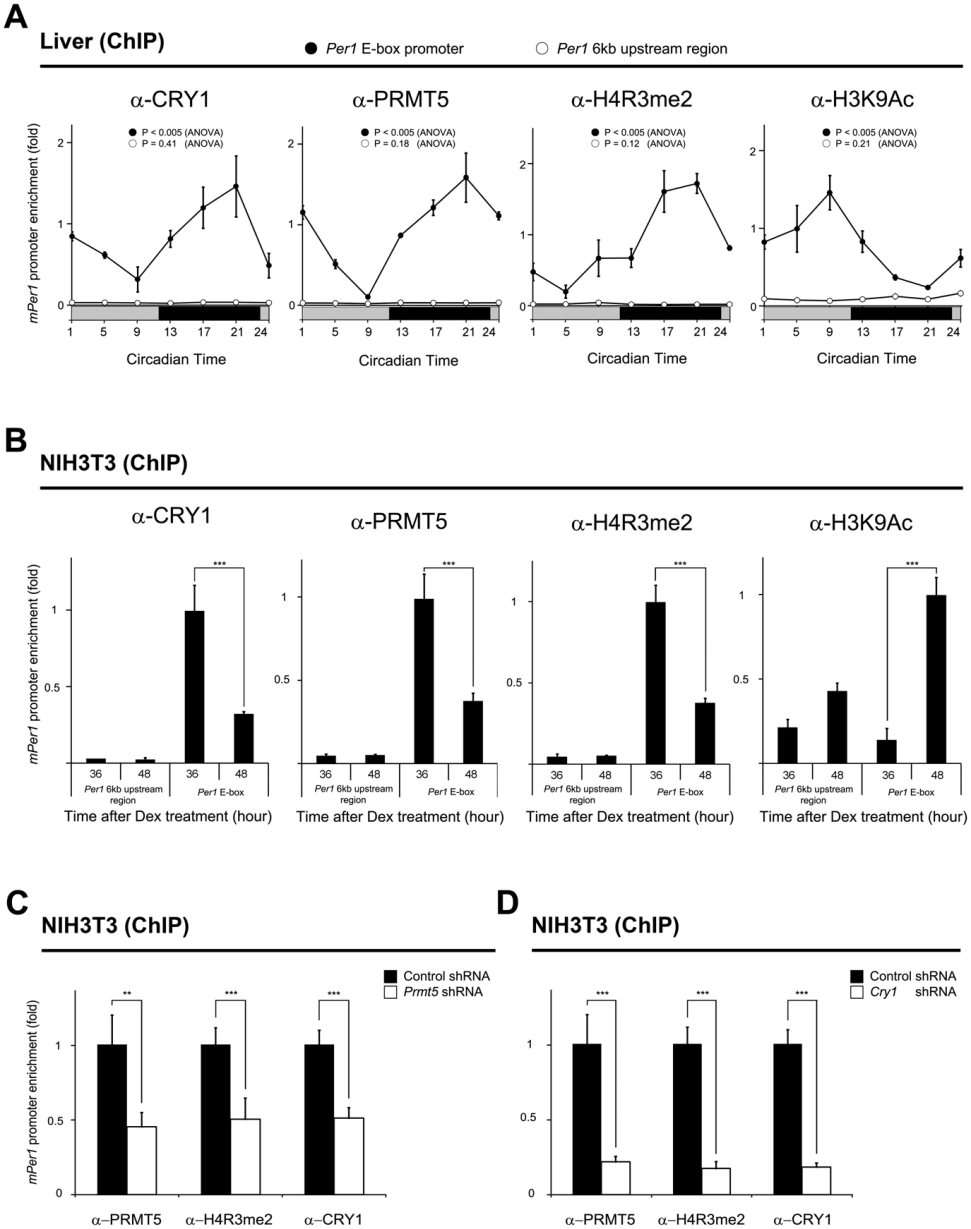
Rhythmic recruitment of PRMT5 to the *Per1* gene promoter coincides with enrichment dimethylated H4R3. A ChIP assay was performed with (A) mouse livers harvested during the circadian cycle using the anti-CRY1 antibody, anti-PRMT5 antibody, anti-dimethylated H4R3 antibody, and anti-acetylated H3K9 antibody at the indicated times. Promoter occupancies of each protein were quantified with quantitative PCR. As a negative control, the upstream genomic region which is 6 kb away from the E-box of the *Per1* gene promoter was amplified (*Per1* 6kb upstream region). Data are presented as mean ± S.E.M. of the three biological replicates with two mice analyzed for each group. Analysis of variance (ANOVA) was used for multiple comparisons. (B) NIH3T3 cells that were synchronized with dexamethasone using the anti-CRY1 antibody, anti-PRMT5 antibody, anti-dimethylated H4R3 antibody, and anti-acetylated H4K9 antibody at the indicated times. Data are presented as mean ± S.E.M. of the three biological replicates with two cell plates analyzed for each group. A paired t-test was used for two group comparison. Significance value was *** *P*≤0.005. (C) Knock-down of *Prmt5* results in decreased dimethylation of H4R3 and recruitment of CRY1 to the *Per1* gene promoter. A ChIP assay was performed using the anti-PRMT5 antibody, anti-CRY1 antibody, and anti-dimethylated H4R3 antibody at 36 hours after synchronization in NIH3T3 cells that were transfected with *Prmt5* shRNA. Data are presented as mean ± S.E.M. of the three biological replicates with two cell plates analyzed for each group. A paired t-test was used for two group comparison. Significance values were ** *P*≤0.01 and *** *P*≤0.005. (D) Knock-down of *Cry1* results in decreased recruitment of PRMT5 and dimethylation of H4R3 to the *Per1* gene promoter. A ChIP assay was performed using the anti-CRY1 antibody, anti-PRMT5 antibody, and anti-dimethylated H4R3 antibody at 36 hours after synchronization in NIH3T3 cells that were transfected with *Cry1* shRNA. Data are presented as mean ± S.E.M. of the three biological replicates with two cell plates analyzed for each group. A paired t-test was used for two group comparison. Significance value was *** *P*≤0.005.

CRY1 binds to the CLOCK/BMAL1 heterodimer and represses *Per1* gene expression. Therefore, we next investigated the association of PRMT5 and CRY1 with the *Per1* gene promoter in the mouse liver samples harvested during the circadian cycle. ChIP assay revealed that binding of CRY1 and PRMT5 to the *Per1* gene promoter increased when *Per1* gene expression was repressed at night (Figures 3A and 5A). However, CRY1 and PRMT5 dissociated from the *Per1* gene promoter in the activation phase of gene expression in the daytime. Collectively, the rhythmic recruitment of CRY1 and PRMT5 to the *Per1* gene promoter occurred in an anti-phasic manner with rhythmic *Per1* expression. Since PRMT5 catalyzes the dimethylation of Arg 3 on histone H4 (H4R3me2), which is generally known as a transcription repressive mark, we investigated whether rhythmic PRMT5 recruitment to the *Per1* gene promoter coincides with enrichment of dimethylated H4R3. We found that dimethylation of H4R3 at the *Per1* gene promoter fluctuated in phase with the rhythmic recruitment of PRMT5 (Figures 3A and 5A). As expected, the acetylated histone H3K9 activation mark (H3K9Ac) anti-phasically oscillated with H4R3 dimethylation (Figure 5A). However, we were unable to detect any significant rhythmic enrichment of PRMT5, CRY1, H4R3me2, and H3K9Ac at the upstream genomic region, which is 6 kb away from the E-box of the *Per1* gene promoter. To further confirm our results, we performed the same ChIP assay when *Per1* gene expression was activated or repressed using a synchronized NIH3T3 cell model. As shown in Figure 5B, we again found that increased recruitment of PRMT5 and CRY1 coincided with increased H4R3 dimethylation and decreased H3K9 acetylation at 36 hours after synchronization when the *Per1* gene expression was repressed. In contrast, we observed that decreased binding of PRMT5 and CRY1 to the *Per1* gene promoter was associated with decreased H4R3 dimethylation and increased H3K9 acetylation at 48 hours after synchronization when the *Per1* gene expression was activated (Figures 3B and 5B). We also did not observe significant rhythmic enrichment of PRMT5, CRY1, H4R3me2, and H3K9Ac at the upstream genomic region, which was used as a negative control (Figure 5B). Collectively, these data suggest that rhythmic recruitment of PRMT5 and CRY to the *Per1* gene promoter is associated with the rhythmic dimethylation of H4R3 and *Per1* gene expression.

In order to examine whether depletion of *Prmt5* is paralleled by decreased dimethylated H4R3, we also performed the ChIP assay when PRMT5 and CRY1 were bound strongly to the *Per1* promoter at 36 hours after synchronization (Figure 5C). We observed that the dimethylation level of H4R3 at the promoter significantly decreased. Interestingly, recruitment of CRY1 to the *Per1* gene promoter also significantly decreased (Figure 5C). We further tested whether depletion of *Cry1* affects the recruitment of PRMT5 by ChIP assay at 36 hours after synchronization (Figure 5D). Consistent with up-regulation of *Per1* gene expression in *Cry1*-depleted cells, we observed significantly decreased enrichment of PRMT5 and H4R3me2 at the promoter (Figures 5D and S3A). These results indicate that both PRMT5 and CRY1 may be required for the repression of the *Per1* gene. When *Cry2* was depleted by shRNA transfection, we observed less pronounced up-regulation of *Per1* gene expression and less decreased enrichment of PRMT5 and H4R3me2 at the promoter compared to *Cry1* depletion (Figure S3).

### Depletion of *Prmt5* causes altered rhythmic expression of the *Per1* gene

**Figure 6:**
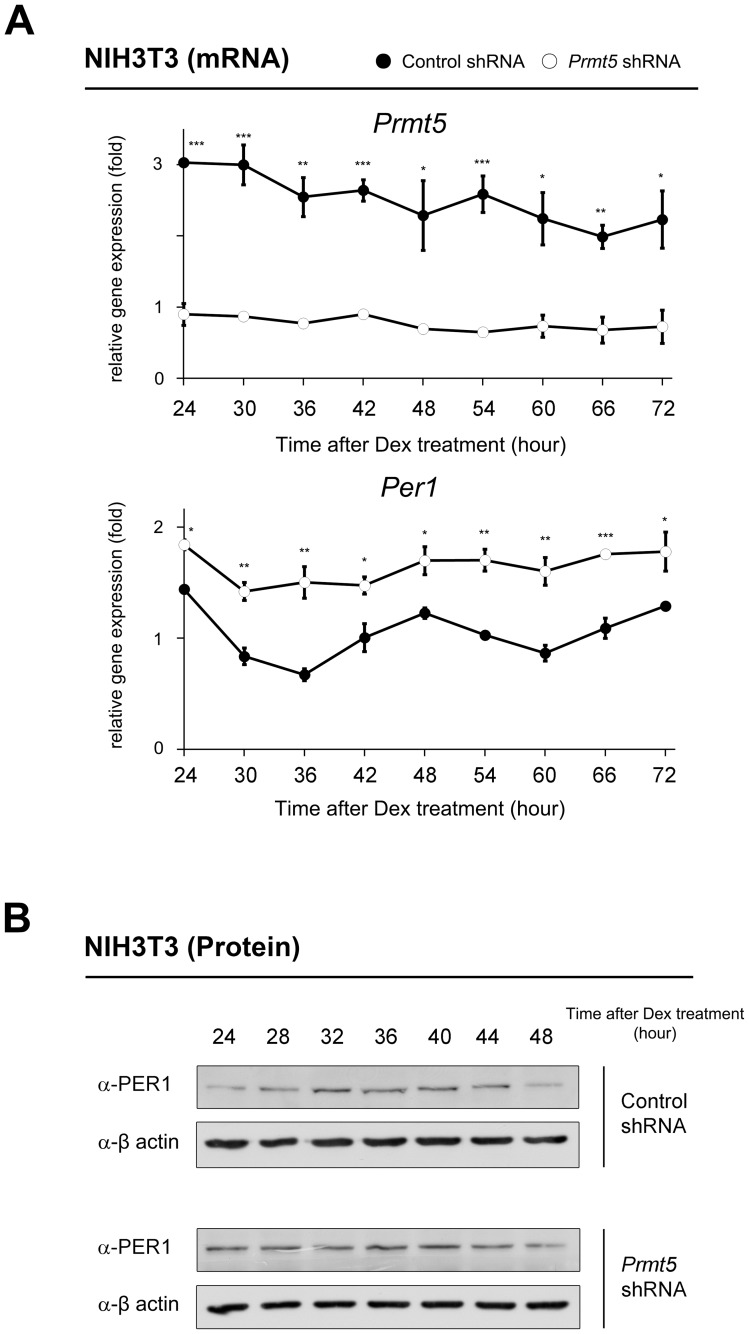
Depletion of *Prmt5* deregulates rhythmic *Per1* expression. (A) Knock-down of *Prmt5* induces up-regulated and non-rhythmic *Per1* gene expression. NIH3T3 cells were transfected with *Prmt5* shRNA and then cells were synchronized with 100 nM dexamethasone for 2 hours. Transcripts of *Prmt5*, *Per1*, and *Gapdh* were measured using quantitative PCR at indicated time. Data are presented as mean ± S.E.M. of the three biological replicates with two cell plates analyzed for each group. A paired t-test was used for two group comparison. Significance values were * *P*≤0.05, ** *P*≤0.01, and *** *P*≤0.005 compared to level of *Prmt5* or *Per1* gene expression at each time point. (B) Knock-down of *Prmt5* induces up-regulated and non-rhythmic PER1 expression at the protein level. NIH3T3 cells were transfected with *Prmt5* shRNA, and then cells were synchronized with 100 nM dexamethasone for 2 hours. Western blot analysis was performed using anti-PER1 and anti-β actin antibodies at the indicated times.

Next, we hypothesized that the depletion of *Prmt5* deregulates expression of the *Per1* gene in the synchronized NIH3T3 cells. After transfection of *Prmt5* shRNA, NIH3T3 cells were synchronized with dexamethasone treatment. As shown in Figure 6A, the *Per1* gene expression was significantly up-regulated. Moreover, rhythmic expression of the *Per*1 gene was abolished in *Prmt5*-depleted cells. We further observed up-regulation of PER1 at the protein level without rhythmicity in *Prmt5*-depleted cells (Figure 6B).

In conclusion, our results consistently support the hypothesis that PRMT5 functions as a transcriptional corepressor in CRY-mediated circadian *Per1* gene regulation through the regulation of H4R3 dimethylation at the promoter.

## Discussion

Regulation of histone modifications such as acetylation and methylation on specific lysine residues plays a crucial role in the gene expression that underlies circadian rhythm. In fact, rhythmic p300-mediated H3 acetylation has been reported at various circadian genes [23]. CRY1 negatively regulates *Per1* gene expression by recruiting histone deacetylases (HDACs) [20]. Rhythmic enrichment of trimethylation of H3K4 and dimethylation of H3K9 were observed at the *Rev-erbα* gene promoter [24]. Dimethylation of histone H3K9 and recruitment of HP1α to the *Dbp* (*albumin D site-binding protein*) gene promoter might be the general mechanism for CRY-mediated transcription repression [21]. In addition, Polycomb group EZH2 regulates di- and tri-methylation of H3K27 at the *Per1* and *Per2* promoters [25]. Recently, it has been proposed that H4K4 trimethylation by MLL1 methyltransferase, which forms complex with CLOCK-BMAL1 is required for circadian transcription [26]. Histone demethylase JARID1a also plays an important role in circadian transcription by inhibiting HDAC1 [27]. In this study, we suggest that PRMT5 interacts with CRY1 and is involved in circadian *Per1* gene expression through the rhythmic arginine dimethylation of H4R3.

PRMT5 is a type II arginine methyltransferase that catalyzes the monomethylation and symmetric dimethylation of arginine residues [28], [29]. PRMT5 has multiple functions including transcriptional regulation, alternative splicing, apoptosis, and ribosome biogenesis. As a splicing regulator, PRMT5 mediates RNA splicing by methylation of Sm proteins, components of the spliceosome [30]. The *Arabidopsis* homolog of *PRMT5* has been found to be important in regulating pre-mRNA splicing [31]. In addition, PRMT5 functions as a transcriptional regulator in a variety of mechanisms. For example, PRMT5-mediated methylation of transcriptional elongation factor SPT5 results in alteration of RNA polymerase II-dependent gene expression [32]. PRMT5 has also been demonstrated to act as a transcriptional corepressor based on its dimethylation activity on H3R8 and H4R3 [33], [34], [35], [36], [37], [38], [39], [40]. Recently, two reports demonstrated that PRMT5 is involved in *Arabidopsis* and *Drosophila* circadian clock regulation. Hong et al. found that PRMT5 is a critical determinant of period length in *Arabidopsis* [41]. Moreover, Sanchez et al. found that PRMT5 is required for the control of alternative splicing of clock genes such as *PRR9* in *Arabidopsis* [42]. They further showed that a *Drosophila PRMT5* homologue (*dart5-1*) is associated with alterations in splicing of the *period* and several clock-associated genes, indicating that PRMT5 links the circadian clock to the regulation of alternative splicing.

Although we cannot exclude the possibility that mammalian PRMT5 also regulates alternative splicing of circadian clock genes, our results indicate that mammalian PRMT5 may act as a transcriptional corepressor in circadian *Per1* gene regulation. First, we found that PRMT5 interacts with the CRY1 transcriptional repressor. In addition, overexpression of PRMT5 augmented the transcriptional repression activity of CRY1 in the reporter assay. Consistent with our finding, the presence of an unknown transcriptional corepressor that binds to the C-terminal region of CRY1 had been postulated because its deletion abolished target gene repression [43]. Second, rhythmic association of PRMT5 with CRY1 at the *Per1* gene promoter coincided with rhythmic dimethylation of H4R3 and transcriptional repression of the *Per1* gene. More importantly, we observed that depletion of *Prmt5* results in a significant reduction of H4R3 dimethylation and alteration of rhythmic *Per1* gene expression.

Although more studies are required to discover species-specific roles of PRMT5, we expect that our observations may have resulted from the different structures of CRYs, since the amino acid composition and length of the C-terminal region of mammalian CRY are quite different from that of *Arabidopsis* and *Drosophila* CRY [44], [45]. In addition, some species-specific mechanisms of circadian clock regulation by CRY have been previously suggested [46]. For example, *Arabidopsis* and *Drosophila* CRY is a major circadian photoreceptor for light entrainment, while mammalian CRY is not required for photo-entrainment.

In this study, we also observed decreased enrichment of CRY1 at the *Per1* gene promoter in *Prmt5*-depleted cells. These results imply that PRMT5 may regulate CRY1 translocation to the nucleus and/or its degradation. In fact, there is speculation that PRMT5 may regulate the nuclear cytoplasmic shuttling of several proteins such as NF-AT family members [47]. Moreover, it has recently been reported that methylation of RAF proteins by PRMT5 is involved in the degradation of RAF proteins in the RAS signaling pathway [48]. Similarly, we observed up-regulation of *Per1* gene expression and decreased enrichment of PRMT5 and H4R3me2 at the promoter in *Cry1*-depleted cells. In contrast, a less pronounced effect of *Cry2* depletion on *Per1* gene regulation was observed, implying that CRY1 may have a major role in PRMT5-mediated *Per1* gene repression. Supporting our observations, several reports indicate that CRY1 may function as the main transcriptional repressor in CLOCK/BMAL1-mediated transcription [49], [50], [51]. However, we do not exclude the possibility that both CRY1 and CRY2 may be required for the full repression of the *Per1* gene.

When we overexpressed Flag-tagged CRY1 (aa 370 to 470) or Flag-tagged CRY1 (aa 471 to 586) in 293T cells, each of the CRY1 fragments formed a homodimer (Figures S1D and S1E). Based on our observation, there is a possibility that PRMT5 interacts with CRY1 when it forms a homodimer. However, we did not detect homodimerization of full-length CRY1 in standard Western blot analysis. Although it is unclear whether homodimerization of each of CRY1 fragment *in vitro* has a physiological impact on PRMT5 interaction or circadian rhythm, it has been previously reported that homodimerization of the N-terminal of the *Arabidopsis* cryptochrome photoreceptor plays an important role in light signaling [52].

In summary, our study demonstrates that PRMT5 interacts with CRY1 *in vivo*. Although non-rhythmic expression of the *Prmt5* gene was observed, rhythmic recruitment of CRY1 and PRMT5 to the *Per1* gene promoter coincides with the rhythmic dimethylation of histone H4R3 and *Per1* gene repression. Consistently, we found decreased H4R3 dimethylation and alteration of rhythmic *Per1* gene expression in *Prmt5*-depleted cells. Our study provides an insight into the link between histone modification by PRMT5 and mammalian circadian clock gene *Per1* regulation.

## Materials and Methods

### Cell culture and animal handling

NIH3T3 and 293T cells were maintained in DMEM supplemented with 10% fetal bovine serum and antibiotics. To synchronize NIH3T3 cells, they were kept for 2–3 days until the cells reached confluence, and then were treated with 100 nM dexamethasone. Two hours after treatment, the medium was replaced with DMEM. At the indicated times, the cells were washed twice with ice-cold phosphate-buffered saline (PBS) and harvested for RNA and protein extraction. For the ChIP assay, the cells were fixed with formaldehyde in PBS (1% final concentration) for 15 min. The committee for experimental animal research at Sogang University approved the animal experiments. Male BALB/c mice (Samtaco, Osan, Korea) were maintained for 2 weeks on a 12 hr: 12 hr light-dark cycle and then released to constant darkness. On the third day in constant darkness, the livers were harvested at the indicated times and kept frozen at −80C until use. For the ChIP assay, the livers were chopped into small pieces and fixed with formaldehyde in PBS (1% final concentration) for 15 min at room temperature. To stop the crosslinking, 1.25 M glycine was added (1% final concentration) for 5 min. Tissues were stored in liquid N_2_ until use.

### Antibodies

The following commercially available antibodies were used: anti-CRY1 antibody (sc-33177, Santa Cruz Biotechnology, Santa Cruz, CA, USA; ab54649, Abcam, Cambridge, MA, USA), anti-PRMT5 antibody (07–405, Millipore, Temecula, CA, USA), anti-dimethyl H4R3 antibody (ab5823, Abcam), anti-acetylated H3K9 antibody (ab4441, Abcam), anti-H4 antibody (05–858, Millipore), anti-β actin antibody (A5471, Sigma-Aldrich, St. Louis, MO, USA), anti-Flag antibody (F1804, Sigma-Aldrich), and anti-HA antibody (MMS101P, Covance, Berkeley, CA, USA). Normal IgG (sc-2027, Santa Cruz Biotechnology) was used as a control.

### Transfection and immunoprecipitation

NIH3T3 cells were transfected using a Lipofectamine 2000 (Invitrogen, Carlsbad, CA, USA) or a Vivamagic transfection reagent (Vivagen, Seongnam, Korea). The 293T cells were transfected using a calcium phosphate method. For immunoprecipitation, cells or tissues were rinsed in PBS, harvested, and sonicated in an IP150 buffer (10% glycerol, 0.5 mM EDTA, 0.1% NP40, 150 mM NaCl, 1 mM DTT, 25 mM Tris, pH 8.0) in the presence of complete protease inhibitors (Roche, Mannheim, Germany) and 1 mM phenylmethylsulphonylfluoride. To isolate the nucleus, cells or tissues were lysed in a nuclei buffer (1.5 mM MgCl_2_, 10 mM KCl, 10 mM HEPES-KOH, pH 7.9). After centrifugation, the nuclear pellet was lysed in an IP150 buffer using sonication. After clearing by centrifugation, the extracts were incubated with the specific antibody overnight at 4°C, followed by incubation with protein A/G agarose beads (Sigma-Aldrich), washed extensively and dissolved in an SDS sample buffer. Western blotting was carried out using standard procedures. Protein concentration was determined by the Lowry method. To detect the monomeric form of CRY1 fragments, proteins were extracted with an SDS sample buffer containing 4 M urea and electrophoresis was performed using SDS-polyacrylamide gel containing 4 M urea.

### Luciferase activity assay

NIH3T3 cells were transfected with various plasmid constructs such as 1.8 kb mouse *Per1* gene promoter-driven firefly luciferase, *thymidine kinase* promoter-driven renilla luciferase, *Myc-Clock, Myc-Bmal1, Flag-Cry1*, and *HA-Prmt5*. After 48 hours, cells were harvested for luciferase activity using the Dual-Luciferase Assay System (Promega, Madison, WI, USA) with a Lumat BL 9507 luminometer (Berthold technologies, Bad Wildbad, Germany). As a negative control, the *Per1* promoter luciferase-reporter containing mutated E-box was used [53], [54]. Renilla luciferase activity served as an internal control for normalizing firefly luciferase activity of the *Per1* gene promoter. All constructs were confirmed by DNA sequencing.

### RNA isolation and quantitative PCR analysis

Total RNA was extracted from cells or tissues using the Nucleospin RNA isolation kit (Macherey-Nagel, Düren, Germany). First-strand cDNA synthesis from the total RNA template was performed with the PrimeScript II 1st strand cDNA Synthesis Kit (Takara, Japan). The resulting cDNAs were subjected to real-time PCR with a Stratagene Mx3000P (Agilent Technologies, Waldbronn, Germany) using the SYBR Green I Mix (Takara, Japan). PCR conditions used to amplify all genes were 30 s at 95°C, 40 cycles of 95°C for 5 s, and 60°C for 34 s. Expression data were calculated from the cycle threshold (Ct) value using the ΔCt method of quantification. *GAPDH* mRNA levels were used for normalization [55]. Amplification was performed using the following primers: 5′-CTGCAACATTCCCAGTACAACCAAGCG-3′ and 5′-ATCAGAGGCTGAAGAGGCAGTGT-3′ for *Per1*, 5′-GTCTTCTCGCCTCGGTCCCTTCTAACT-3′ and 5′-CCATTCCCGCTGCTGCTACAAC-3′ for mouse *Cry 1*, 5′-TGTGGTGGCATAACTTTCGGACTCTGT-3′ and 5′-TGGGGAGAATGGCTGCTTTGAT-3′ for mouse *Prmt5*, and 5′-AGGTCGGTGTGAACGGATTTG-3′ and 5′-TGTAGACCATGTAGTTGAGGTCA-3′ for mouse *Gapdh*. For validation of all PCR primers, quantitative PCR amplification plots and dissociation curves were analyzed and a single band of the expected size was confirmed after PCR by agarose gel analysis.

### Chromatin immunoprecipitation (ChIP)

NIH3T3 cells or tissues were collected and crosslinked using 1% formaldehyde in PBS for 15 min at room temperature. To stop the crosslinking, 1.25 M glycine was added (1% final concentration). After washing with ice-cold PBS, the cells in a SDS lysis buffer (1% SDS, 10 mM EDTA, 50 mM Tris-HCl, pH 8.1) were sonicated until the DNA fragments were 300–500 bp in size. The extracts were subsequently centrifuged, and the resulting soluble chromatin solutions were diluted ten fold with a ChIP dilution buffer (0.01% SDS, 1.1% Triton X-100, 1.2 mM EDTA, 167 mM NaCl, 16.7 mM Tris-HCl, pH 8.1). The specific antibodies or IgG were added to a soluble chromatin solution overnight at 4°C. Protein A-sepharose beads (Sigma-Aldrich) were further added to the solution in order to precipitate the DNA-protein complexes for 2 hr. The beads were extensively washed with a low salt wash buffer (0.1% SDS, 1% Triton X-100, 2 mM EDTA, 150 mM NaCl, 20 mM Tris-HCl, pH 8.1), a high salt wash buffer (0.1% SDS, 1% Triton X-100, 2 mM EDTA, 500 mM NaCl, 20 mM Tris-HCl, pH 8.1), an LiCl wash buffer (0.25 M LiCl, 1% NP-40, 1% deoxycholate, 1 mM EDTA, 10 mM Tris-HCl, pH 8.1), and finally with a TE buffer. After elution of the DNA/protein with 1% SDS, crosslinking was reversed for 6 hr at 65°C. The DNA was recovered using the QIAquick spin column (Qiagen, Valencia, CA, USA). Real time-PCR was performed with a Stratagene Mx3000P using primers that cover the mouse *Per1* promoter (5′-ACGGTGTGAGACATCCTGATCGCATTG-3′ and 5′-TCTTCCTGGCATCTGATTGGCTACTGG -3′). As a negative control, the upstream genomic region which is 6 kb away from the E-box of the mouse *Per1* gene promoter, was amplified using primers (5′- TCCCGAACTCTTGAACTG-3′ and 5′- GCAACCAGAAATGCTACC-3′). Briefly, 1–2 μl of immunoprecipitated DNA was used as a template in 20 μl reactions containing 1× SYBR Green Master Mix (Takara).

All PCR reactions were performed in triplicate and included negative controls (IgG) as well as positive controls (genomic DNA). The relative proportions of immunoprecipitated fragments were determined using the ΔCt comparative method, based on the threshold cycle (Ct) value for each PCR reaction, and normalized to input genomic DNA. For validation of all PCR primers, quantitative PCR amplification plots and dissociation curves were analyzed, and a single band of the expected size was confirmed after PCR by agarose gel analysis.

### RNA interference (siRNA/shRNA)

NIH3T3 cells were transfected with siRNA (M-042281-01-0005, Dharmacon, Lafayette, CO, USA) or shRNA (TRCN0000181891, Sigma-Aldrich) against *Prmt5*. To knock down *Cry1* and *Cry2*, shRNAs (TRCN0000173481 and TRCN0000194121, Sigma-Aldrich) were used, respectively. For a control, siRNA (sc37007, Santa Cruz Biotechnology) or shRNA (SHC002, Sigma-Aldrich) was used. The efficiency of the knock down of specific genes was confirmed with real time-PCR.

## Supporting Information

Figure S1 **Confirmation of the interaction between CRY1 and PRMT5.** (A) The lysates from overexpressed untagged PRMT5 and CRY1 in 293T cells were immunoprecipitated with the anti-Flag antibody and immunoblotted with the anti-HA antibody or (B) immunoprecipitated with the anti-HA antibody and immunoblotted with the anti-Flag antibody. Inputs were immunoblotted with anti-CRY1 and anti-PRMT5 antibodies. IgG was used as the negative control. (C) CRY1 did not interact with NF-κB (p65). The lysates from overexpressed Flag-tagged CRY1 and HA-tagged NF-κB (p65) in 293T cells were immunoprecipitated with the anti-Flag antibody and immunoblotted with the anti-HA antibody (left) or immunoprecipitated with the anti-HA antibody and immunoblotted with the anti-Flag antibody (right). IgG was used as the negative control. (D) Confirmation of overexpressed CRY1 fragments. The lysates from the 293T cells overexpressing Flag-tagged CRY1 (aa 1 to 374), Flag-tagged CRY1 (aa 370 to 470), and Flag-tagged CRY1 (aa 471 to 586) were immunoblotted with the anti-CRY1 antibody, which can detect the entire region of CRY1 (left). (E) Evidence for the homodimerization of CRY1 fragments. After each Flag-tagged CRY1 (aa 370 to 470) and Flag-tagged CRY1 (aa 471 to 586) was overexpressed in 293T cells, the cell extracts preparation and SDS-PAGE were performed in the presence of 4 M urea. Western blot analysis was performed using anti-Flag (left) or anti-CRY1 antibodies (right). Note that the expected molecular weight of CRY1 fragments was observed. (F) The specificity of interaction between CRY1 and PRMT5 was confirmed using *Cry1*-depleted cells. The lysates from *Cry1* shRNA-transfected NIH3T3 cells at 36 hours after synchronization were immunoprecipitated with the anti-PRMT5 antibody and immunoblotted with the anti-CRY1 and anti-PRMT5 antibodies. IgG was used as the negative control. (G) The specificity of interaction between CRY1 and PRMT5 was confirmed using *Prmt5*-depleted cells. The lysates from *Prmt5* shRNA-transfected NIH3T3 cells at 36 hours after synchronization were immunoprecipitated with the anti-PRMT5 antibody and immunoblotted with the anti-PRMT5 and anti-CRY1 antibodies. IgG was used as the negative control.
(TIF)

Figure S2 **Confirmation of antibody specificity.** (A) The lysates from control shRNA or *Cry1* shRNA-transfected NIH3T3 cells were immunoblotted with the anti-CRY1 antibody. The anti-β actin antibody was used as a loading control. (B) The lysates from control shRNA or *Prmt5* shRNA-transfected NIH3T3 cells were immunoblotted with the anti-PRMT5 antibody. The anti-β actin antibody was used as a loading control. (C) The lysates from control shRNA or *Prmt5* shRNA-transfected NIH3T3 cells were immunoblotted with the anti-H4R3me2 antibody. The anti-histone H4 antibody was used as a loading control.
(TIF)

Figure S3 **Depletion of** ***Cry*** **deregulates rhythmic** ***Per1*** **gene expression.** (A) Knock-down of *Cry1* induces up-regulated and non-rhythmic *Per1* gene expression. NIH3T3 cells were transfected with *Cry1* shRNA and then cells were synchronized with 100 nM dexamethasone for 2 hours. Transcripts of *Per1, Cry1*, and *Gapdh* were measured using quantitative PCR at 36 hours after synchronization. Data are presented as mean ± S.E.M. of the three biological replicates with two cell plates analyzed for each group. A paired t-test was used for two group comparison. Significance value was *** *P*≤0.005. (B) Knock-down of *Cry1* results in significantly decreased enrichment of PRMT5 and H4R3me2 at the *Per1* gene promoter. A ChIP assay was performed using the anti-CRY1 antibody, anti-PRMT5 antibody, and anti-dimethylated H4R3 antibody at 36 hours after synchronization in NIH3T3 cells that were transfected with *Cry1* shRNA. Data are presented as mean ± S.E.M. of the three biological replicates with two cell plates analyzed for each group. A paired t-test was used for two group comparison. Significance value was *** *P*≤0.005. (C) Knock-down of *Cry2* shows less pronounced effect on the *Per1* gene expression. NIH3T3 cells were transfected with *Cry2* shRNA and then cells were synchronized with 100 nM dexamethasone for 2 hours. Transcripts of *Per1, Cry2*, and *Gapdh* were measured using quantitative PCR at 36 hours after synchronization. Data are presented as mean ± S.E.M. of the three biological replicates with two cell plates analyzed for each group. A paired t-test was used for two group comparison. Significance value was *** *P*≤0.005. (D) Knock-down of *Cry2* results in less decreased enrichment of PRMT5 and H4R3me2 at the *Per1* gene promoter. A ChIP assay was performed using the anti-CRY1 antibody, anti-PRMT5 antibody, and anti-dimethylated H4R3 antibody at 36 hours after synchronization in NIH3T3 cells that were transfected with *Cry2* shRNA. Data are presented as mean ± S.E.M. of the three biological replicates with two cell plates analyzed for each group. A paired t-test was used for two group comparison. Significance values were * *P*≤0.05, ** *P*≤0.01, and *** *P*≤0.005. Figure 5D was designated as Figure S3B to more conveniently compare the effect of *Cry1* and *Cry2* depletion on *Per1* gene expression.
(TIF)

Table S1 **Proteins interacting with CRY1 by mass spectrometry.**
(DOCX)
