# Supplementary figures and images for: Role of Type II Protein Arginine Methyltransferase 5 in the Regulation of Circadian Per1 Gene
Source: PLoS One. 2012 Oct 25;7(10):e48152. doi: 10.1371/journal.pone.0048152 (PMC3485018; doi:10.1371/journal.pone.0048152)

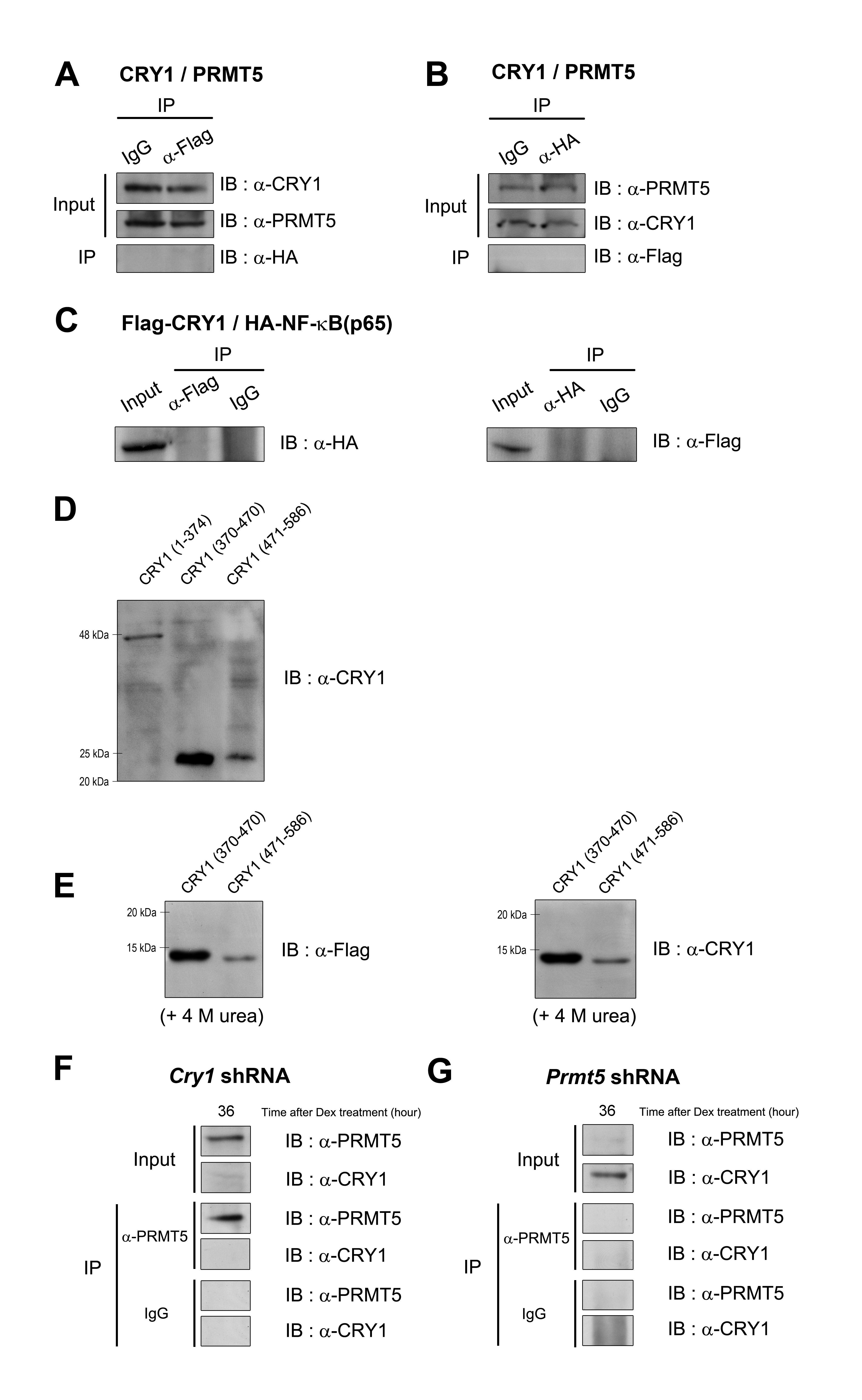

Supplement: Figure S1 — Confirmation of the interaction between CRY1 and PRMT5. (A) The lysates from overexpressed untagged PRMT5 and CRY1 in 293T cells were immunoprecipitated with the anti-Flag antibody and immunoblotted with the anti-HA antibody or (B) immunoprecipitated with the anti-HA antibody and immunoblotted with the anti-Flag antibody. Inputs were immunoblotted with anti-CRY1 and anti-PRMT5 antibodies. IgG was used as the negative control. (C) CRY1 did not interact with NF-κB (p65). The lysates from overexpressed Flag-tagged CRY1 and HA-tagged NF-κB (p65) in 293T cells were immunoprecipitated with the anti-Flag antibody and immunoblotted with the anti-HA antibody (left) or immunoprecipitated with the anti-HA antibody and immunoblotted with the anti-Flag antibody (right). IgG was used as the negative control. (D) Confirmation of overexpressed CRY1 fragments. The lysates from the 293T cells overexpressing Flag-tagged CRY1 (aa 1 to 374), Flag-tagged CRY1 (aa 370 to 470), and Flag-tagged CRY1 (aa 471 to 586) were immunoblotted with the anti-CRY1 antibody, which can detect the entire region of CRY1 (left). (E) Evidence for the homodimerization of CRY1 fragments. After each Flag-tagged CRY1 (aa 370 to 470) and Flag-tagged CRY1 (aa 471 to 586) was overexpressed in 293T cells, the cell extracts preparation and SDS-PAGE were performed in the presence of 4 M urea. Western blot analysis was performed using anti-Flag (left) or anti-CRY1 antibodies (right). Note that the expected molecular weight of CRY1 fragments was observed. (F) The specificity of interaction between CRY1 and PRMT5 was confirmed using Cry1-depleted cells. The lysates from Cry1 shRNA-transfected NIH3T3 cells at 36 hours after synchronization were immunoprecipitated with the anti-PRMT5 antibody and immunoblotted with the anti-CRY1 and anti-PRMT5 antibodies. IgG was used as the negative control. (G) The specificity of interaction between CRY1 and PRMT5 was confirmed using Prmt5-depleted cells. The lysates from Prmt5 shRNA-tra [file pone.0048152.s001.tif]

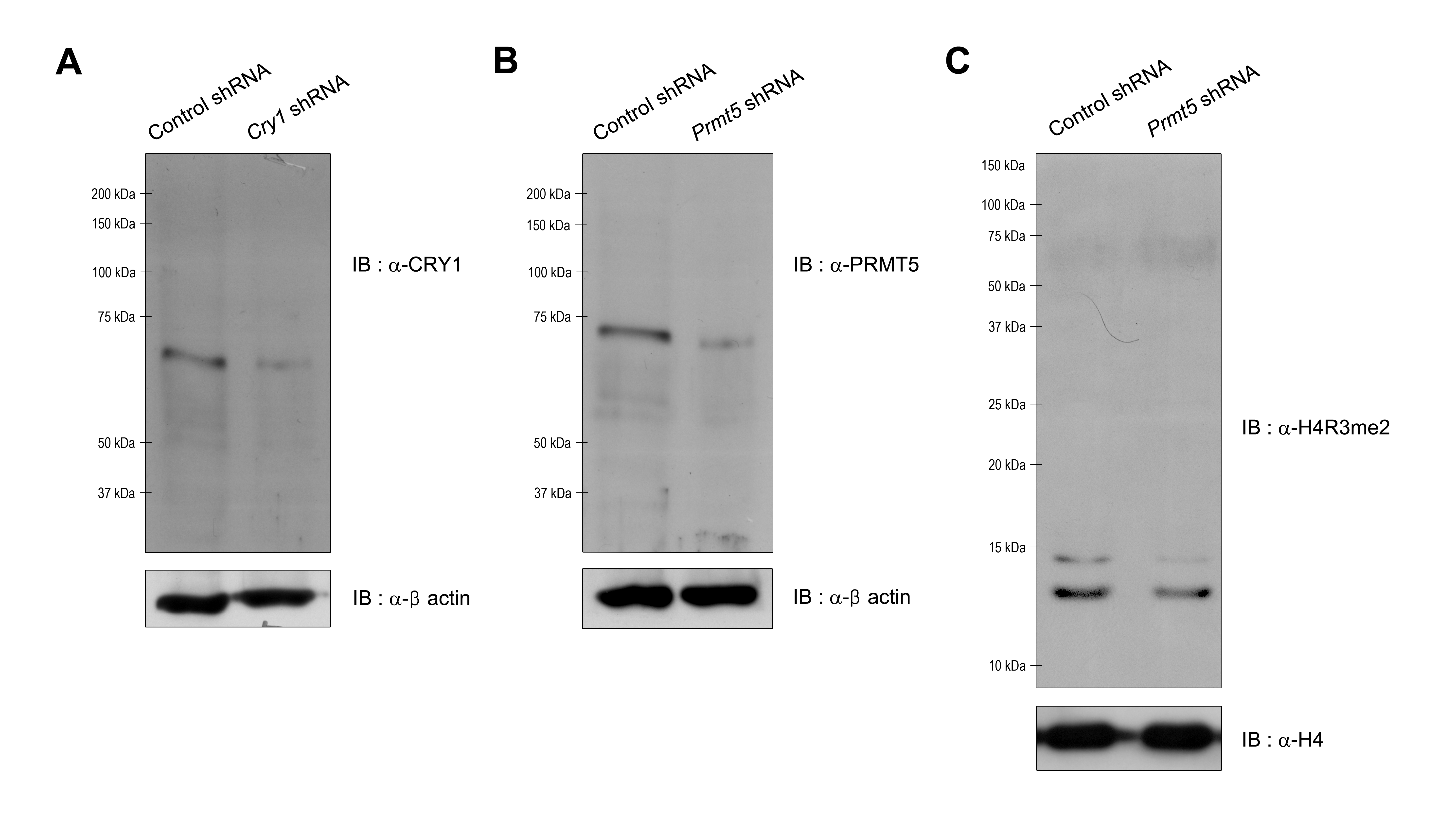

Supplement: Figure S2 — Confirmation of antibody specificity. (A) The lysates from control shRNA or Cry1 shRNA-transfected NIH3T3 cells were immunoblotted with the anti-CRY1 antibody. The anti-β actin antibody was used as a loading control. (B) The lysates from control shRNA or Prmt5 shRNA-transfected NIH3T3 cells were immunoblotted with the anti-PRMT5 antibody. The anti-β actin antibody was used as a loading control. (C) The lysates from control shRNA or Prmt5 shRNA-transfected NIH3T3 cells were immunoblotted with the anti-H4R3me2 antibody. The anti-histone H4 antibody was used as a loading control. (TIF) [file pone.0048152.s002.tif]

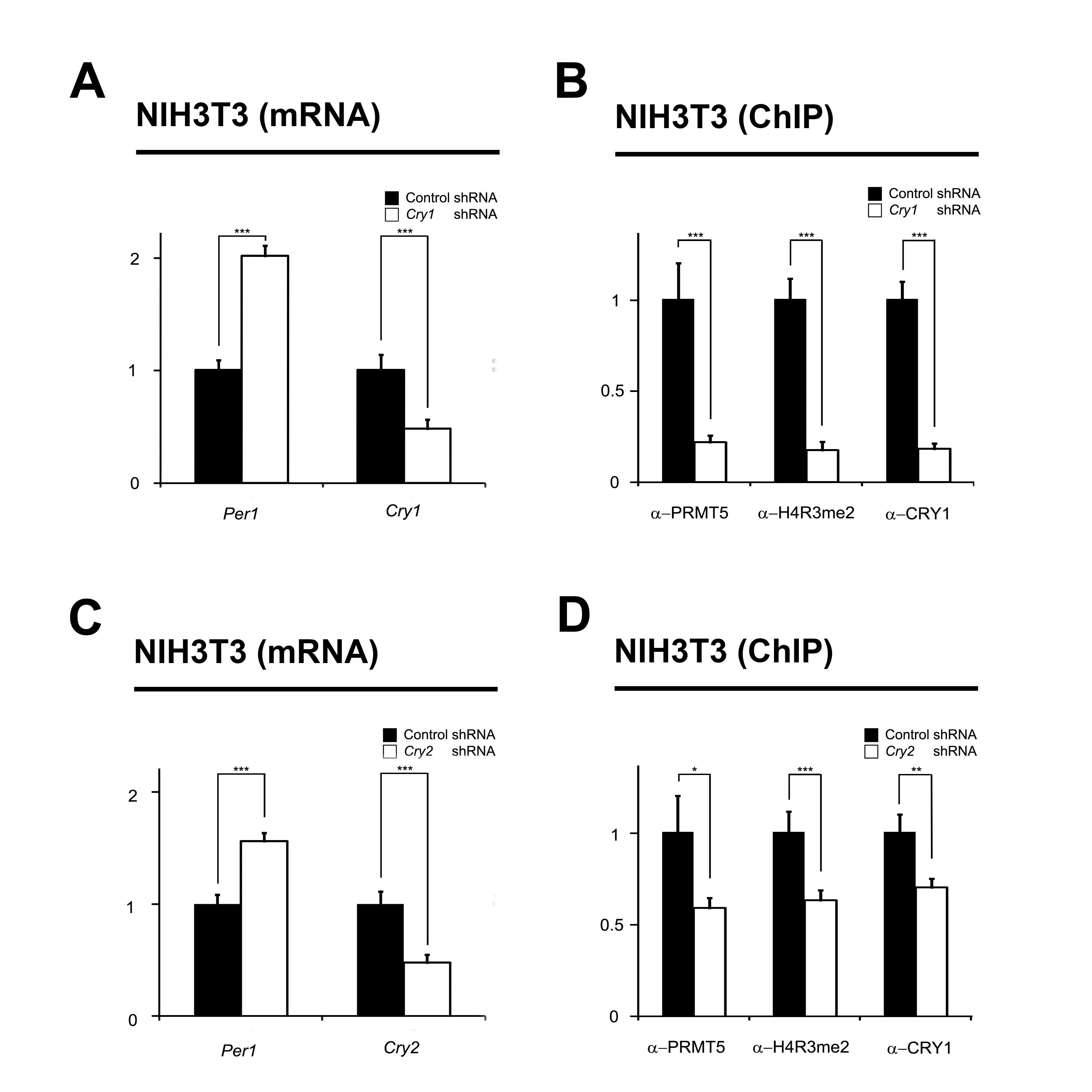

Supplement: Figure S3 — Depletion of Cry deregulates rhythmic Per1 gene expression. (A) Knock-down of Cry1 induces up-regulated and non-rhythmic Per1 gene expression. NIH3T3 cells were transfected with Cry1 shRNA and then cells were synchronized with 100 nM dexamethasone for 2 hours. Transcripts of Per1, Cry1, and Gapdh were measured using quantitative PCR at 36 hours after synchronization. Data are presented as mean ± S.E.M. of the three biological replicates with two cell plates analyzed for each group. A paired t-test was used for two group comparison. Significance value was *** P≤0.005. (B) Knock-down of Cry1 results in significantly decreased enrichment of PRMT5 and H4R3me2 at the Per1 gene promoter. A ChIP assay was performed using the anti-CRY1 antibody, anti-PRMT5 antibody, and anti-dimethylated H4R3 antibody at 36 hours after synchronization in NIH3T3 cells that were transfected with Cry1 shRNA. Data are presented as mean ± S.E.M. of the three biological replicates with two cell plates analyzed for each group. A paired t-test was used for two group comparison. Significance value was *** P≤0.005. (C) Knock-down of Cry2 shows less pronounced effect on the Per1 gene expression. NIH3T3 cells were transfected with Cry2 shRNA and then cells were synchronized with 100 nM dexamethasone for 2 hours. Transcripts of Per1, Cry2, and Gapdh were measured using quantitative PCR at 36 hours after synchronization. Data are presented as mean ± S.E.M. of the three biological replicates with two cell plates analyzed for each group. A paired t-test was used for two group comparison. Significance value was *** P≤0.005. (D) Knock-down of Cry2 results in less decreased enrichment of PRMT5 and H4R3me2 at the Per1 gene promoter. A ChIP assay was performed using the anti-CRY1 antibody, anti-PRMT5 antibody, and anti-dimethylated H4R3 antibody at 36 hours after synchronization in NIH3T3 cells that were transfected with Cry2 shRNA. Data are presented as mean ± S.E.M. of the three biological replicates with two [file pone.0048152.s003.tif]
