# Supplementary material for: Role of Type II Protein Arginine Methyltransferase 5 in the Regulation of Circadian Per1 Gene
Source: PLoS One. 2012 Oct 25;7(10):e48152. doi: 10.1371/journal.pone.0048152 (PMC3485018; doi:10.1371/journal.pone.0048152)
Supplement: Table S1 — Proteins interacting with CRY1 by mass spectrometry. (DOCX) [file pone.0048152.s004.docx]

**Table S1. Proteins interacting with CRY1 by mass spectrometry**

| Protein Name | Accesion # | SCORE* | Remarks |
| --- | --- | --- | --- |
| histone cluster 4, H4 | 28173560 | 60.18 |  |
| cryptochrome 2 (photolyase-like) isoform 2 | 188536103 | 50.30 | PER1 interaction. Lee et al., 2004 |
| heterogeneous nuclear ribonucleoprotein A3 | 34740329 | 50.27 |  |
| interleukin enhancer binding factor 3 isoform e | 212549555 | 50.18 |  |
| nucleophosmin 1 isoform 3 | 83641870 | 40.25 |  |
| polypyrimidine tract-binding protein 1 isoform c | 14165466 | 40.22 |  |
| solute carrier family 25, member 5 | 156071459 | 40.18 |  |
| ribosomal protein L4 | 16579885 | 30.22 |  |
| glyceraldehyde-3-phosphate dehydrogenase | 7669492 | 30.22 |  |
| heat shock 70kDa protein 5 | 16507237 | 30.22 |  |
| karyopherin alpha 2 | 4504897 | 30.21 | CRY2 interaction. Sakakida et al., 2005 |
| MYB binding protein 1a isoform 1 | 157694494 | 30.14 | CRY1 interaction. Hara et al., 2009 |
| complement component 1, q subcomponent binding protein precursor | 4502491 | 20.29 |  |
| nucleolin | 55956788 | 20.28 |  |
| eukaryotic translation elongation factor 1 beta 2 | 11136628 | 20.28 |  |
| heat shock 70kDa protein 9 precursor | 24234688 | 20.24 |  |
| heterogeneous nuclear ribonucleoprotein U isoform a | 74136883 | 20.23 | CRY1 interaction. Hara et al., 2009 |
| ribosomal protein L7a | 4506661 | 20.21 | CRY1 interaction. Hara et al., 2009 |
| DEAD/H (Asp-Glu-Ala-Asp/His) box polypeptide 3 | 87196351 | 20.20 |  |
| ribosomal protein L18 | 4506607 | 20.20 |  |
| DnaJ (Hsp40) homolog, subfamily A, member 1 | 4504511 | 20.18 |  |
| interleukin enhancer binding factor 2 | 24234747 | 20.18 |  |
| chaperonin | 41399285 | 20.18 |  |
| DEAH (Asp-Glu-Ala-His) box polypeptide 9 | 100913206 | 20.18 | PER1 interaction. Padmanabhan et al., 2012 |
| histone cluster 2, H2be | 4504277 | 20.18 |  |
| lamin B1 | 5031877 | 20.18 |  |
| ribosomal protein S3 | 15718687 | 20.17 | CRY1 interaction. Hara et al., 2009 |
| ribosomal protein S18 | 11968182 | 20.17 |  |
| activating signal cointegrator 1 complex subunit 3-like 1 | 40217847 | 20.16 |  |
| ribosomal protein L7 | 15431301 | 20.16 |  |
| pinin, desmosome associated protein | 33356174 | 20.15 | TIMELESS interaction. Gotter, 2003 |
| TAR DNA binding protein | 6678271 | 20.14 |  |
| LUC7-like 2 | 116812577 | 10.26 |  |
| histone cluster 1, H2ai | 4504239 | 10.25 |  |
| heat shock 70kDa protein 1-like | 124256496 | 10.24 |  |
| prohibitin 2 | 221307584 | 10.21 | Casein kinase 1 interaction. Kategaya et al., 2012 |
| karyopherin beta 1 | 19923142 | 10.21 | CRY2 interaction. Sakakida et al., 2005 |
| PREDICTED: similar to hCG2042722 | 169218253 | 10.20 |  |
| BCL2-associated transcription factor 1 isoform 1 | 7661958 | 10.20 |  |
| DEAD (Asp-Glu-Ala-Asp) box polypeptide 5 | 4758138 | 10.19 | PER1 intertaction. Padmanabhan et al., 2012 |
| ribosomal protein L19 | 4506609 | 10.19 |  |
| protein phosphatase 1, catalytic subunit, alpha isoform 3 | 56790945 | 10.19 | PER2 interaction. Gallego et al., 2006 |
| HLA-B associated transcript 5 | 15100151 | 10.19 |  |
| peripherin | 21264345 | 10.18 |  |
| reticulocalbin 2, EF-hand calcium binding domain | 4506457 | 10.18 |  |
| DEAD box polypeptide 17 isoform 4 | 148613858 | 10.18 |  |
| splicing factor proline/glutamine rich (polypyrimidine tract binding protein associated) | 4826998 | 10.18 | PER interaction. Duong et al., 2011 |
| nucleophosmin 1 isoform 2 | 40353734 | 10.18 |  |
| nuclease sensitive element binding protein 1 | 34098946 | 10.17 |  |
| solute carrier family 12, member 8 | 38569457 | 10.17 |  |
| ribosomal protein L23a | 17105394 | 10.17 |  |
| ribosomal protein S2 | 15055539 | 10.17 |  |
| SMAD family member 9 isoform a | 187828357 | 10.16 |  |
| zinc finger CCCH-type containing 18 | 31377595 | 10.16 |  |
| caltractin | 4757902 | 10.16 |  |
| ribosomal protein S5 | 13904870 | 10.16 |  |
| NLR family, pyrin domain containing 11 | 194018484 | 10.16 |  |
| RNA polymerase I subunit isoform 1 | 42560246 | 10.16 |  |
| apoptosis antagonizing transcription factor | 7657013 | 10.16 |  |
| insulin receptor substrate 4 | 4504733 | 10.16 |  |
| histone cluster 1, H2bk | 18105048 | 10.16 |  |
| DnaJ (Hsp40) homolog, subfamily C, member 7 isoform 2 | 221219056 | 10.16 |  |
| protein arginine methyltransferase 5 isoform b | 88900507 | 10.16 | CRY1 interaction, this study |
| protein kinase C, theta | 5453976 | 10.15 |  |
| zinc finger protein 185 | 148298722 | 10.15 |  |
| histone cluster 1, H2ba | 24586679 | 10.15 |  |
| spectrin, alpha, non-erythrocytic 1 (alpha-fodrin) isoform 1 | 194595509 | 10.15 |  |
| shroom family member 3 protein | 203098098 | 10.15 |  |
| PPAR-alpha interacting complex protein 285 isoform 1 | 156105693 | 10.15 |  |
| PREDICTED: hypothetical protein LOC401258 | 169169622 | 10.15 |  |
| capping protein (actin filament) muscle Z-line, alpha 2 | 5453599 | 10.15 |  |
| Ewing sarcoma breakpoint region 1 isoform EWS-b | 7669490 | 10.15 | PER1 interaction. Ravasi et al., 2010 |
| YTH domain containing 2 | 38505213 | 10.15 |  |
| ribosomal protein SA | 59859885 | 10.15 |  |
| xin actin-binding repeat containing 2 isoform 2 | 119372315 | 10.15 |  |
| PREDICTED: hypothetical protein | 169211910 | 10.15 |  |
| hypothetical protein LOC57501 | 149944604 | 10.15 |  |
| ADAM metallopeptidase domain 9 isoform 2 precursor | 54292121 | 10.15 |  |
| eukaryotic translation initiation factor 4B | 50053795 | 10.15 |  |
| activating signal cointegrator 1 complex subunit 2 | 20270253 | 10.15 |  |
| nucleolar protein 1, 120kDa | 76150625 | 10.15 |  |
| phosphodiesterase 6H, cGMP-specific, cone, gamma | 5453868 | 10.15 |  |
| AT rich interactive domain 5A | 47078224 | 10.15 |  |
| BCL-6 interacting corepressor isoform a | 183396783 | 10.15 |  |
| hypothetical protein LOC203238 | 38348729 | 10.15 |  |
| titin isoform N2-A | 110349719 | 10.15 |  |
| interferon-induced protein with tetratricopeptide repeats 1-like | 58330892 | 10.15 |  |
| ribosomal protein S14 | 68160922 | 10.15 |  |
| ribosomal protein S7 | 4506741 | 10.15 |  |

*score; a value that is based upon the probability that the peptide is a random match to the spectral data.

References

[Hara Y](http://www.ncbi.nlm.nih.gov/pubmed?term=Hara%20Y%5BAuthor%5D&cauthor=true&cauthor_uid=19129230), [Onishi Y](http://www.ncbi.nlm.nih.gov/pubmed?term=Onishi%20Y%5BAuthor%5D&cauthor=true&cauthor_uid=19129230), [Oishi K](http://www.ncbi.nlm.nih.gov/pubmed?term=Oishi%20K%5BAuthor%5D&cauthor=true&cauthor_uid=19129230), [Miyazaki K](http://www.ncbi.nlm.nih.gov/pubmed?term=Miyazaki%20K%5BAuthor%5D&cauthor=true&cauthor_uid=19129230), [Fukamizu A](http://www.ncbi.nlm.nih.gov/pubmed?term=Fukamizu%20A%5BAuthor%5D&cauthor=true&cauthor_uid=19129230), et al. (2009) Molecular characterization of Mybbp1a as a co-repressor on the Period2 promoter. [Nucleic Acids Res](http://www.ncbi.nlm.nih.gov/pubmed?term=hara%2C%20cry1) 37:1115-1126.

Padmanabhan K, Robles MS, Westerling T, Weitz CJ (2012) [Feedback Regulation of Transcriptional Termination by the Mammalian Circadian Clock PERIOD Complex.](http://www.ncbi.nlm.nih.gov/pubmed/22767893) Science DOI:10.1126

[Lee C](http://www.ncbi.nlm.nih.gov/sites/entrez?db=pubmed&cmd=Search&itool=pubmed_Abstract&term=Lee%20C%5bAUTHOR%5d#_blank), [Weaver DR](http://www.ncbi.nlm.nih.gov/sites/entrez?db=pubmed&cmd=Search&itool=pubmed_Abstract&term=Weaver%20DR%5bAUTHOR%5d#_blank), [Reppert SM](http://www.ncbi.nlm.nih.gov/sites/entrez?db=pubmed&cmd=Search&itool=pubmed_Abstract&term=Reppert%20SM%5bAUTHOR%5d#_blank) (2004) Direct association between mouse PERIOD and CKIepsilon is critical for a functioning circadian clock. **Mol Cell Biol** 24:84-94.

Sakakida Y, Miyamoto Y, Nagoshi E, Akashi M, Nakamura TJ, et al. (2005) [Importin alpha/beta mediates nuclear transport of a mammalian circadian clock component, mCRY2, together with mPER2, through a bipartite nuclear localization signal.](http://www.ncbi.nlm.nih.gov/pubmed/15689618) J Biol Chem 80:13272-13278.

Gotter AL (2003) [Tipin, a novel timeless-interacting protein, is developmentally co-expressed with timeless and disrupts its self-association.](http://www.ncbi.nlm.nih.gov/pubmed/12875843) J Mol Biol 331:167-176.

[Kategaya LS](http://www.ncbi.nlm.nih.gov/pubmed?term=Kategaya%20LS%5BAuthor%5D&cauthor=true&cauthor_uid=22384121), [Hilliard A](http://www.ncbi.nlm.nih.gov/pubmed?term=Hilliard%20A%5BAuthor%5D&cauthor=true&cauthor_uid=22384121), [Zhang L](http://www.ncbi.nlm.nih.gov/pubmed?term=Zhang%20L%5BAuthor%5D&cauthor=true&cauthor_uid=22384121), [Asara JM](http://www.ncbi.nlm.nih.gov/pubmed?term=Asara%20JM%5BAuthor%5D&cauthor=true&cauthor_uid=22384121), [Ptáček LJ](http://www.ncbi.nlm.nih.gov/pubmed?term=Pt%C3%A1%C4%8Dek%20LJ%5BAuthor%5D&cauthor=true&cauthor_uid=22384121), et al. (2012) Casein kinase 1 proteomics reveal prohibitin 2 function in molecular clock. [PLoS One](http://www.ncbi.nlm.nih.gov/pubmed?term=Casein%20kinase%2C%20Kategaya) 7:e31987.

Gallego M, Kang H, Virshup DM (2006) [Protein phosphatase 1 regulates the stability of the circadian protein PER2.](http://www.ncbi.nlm.nih.gov/pubmed/16813562) Biochem J 399:169-175.

Ravasi T, Suzuki H, Cannistraci CV, Katayama S, Bajic VB, et al. (2010) [An atlas of combinatorial transcriptional regulation in mouse and man.](http://www.ncbi.nlm.nih.gov/pubmed/20211142) Cell 140:744-752.

Duong HA, Robles MS, Knutti D, Weitz CJ (2011) [A molecular mechanism for circadian clock negative feedback.](http://www.ncbi.nlm.nih.gov/pubmed/21680841) Science 332:1436-1439.
